# Adjustments of Protein Metabolism in Fasting Arctic Charr, *Salvelinus alpinus*

**DOI:** 10.1371/journal.pone.0153364

**Published:** 2016-04-20

**Authors:** Alicia A. Cassidy, Roxanne J. Saulnier, Simon G. Lamarre

**Affiliations:** Département de biologie, Université de Moncton, Moncton, NB, Canada; Universitat de Barcelona, SPAIN

## Abstract

Protein metabolism, including the interrelated processes of synthesis and degradation, mediates the growth of an animal. In ectothermic animals, protein metabolism is responsive to changes in both biotic and abiotic conditions. This study aimed to characterise responses of protein metabolism to food deprivation that occur in the coldwater salmonid, Arctic charr, *Salvelinus alpinus*. We compared two groups of Arctic charr: one fed continuously and the other deprived of food for 36 days. We measured the fractional rate of protein synthesis (*K*_*S*_) in individuals from the fed and fasted groups using a flooding dose technique modified for the use of deuterium-labelled phenylalanine. The enzyme activities of the three major protein degradation pathways (ubiquitin proteasome, lysosomal cathepsins and the calpain systems) were measured in the same fish. This study is the first to measure both *K*_*S*_ and the enzymatic activity of protein degradation in the same fish, allowing us to examine the apparent contribution of different protein degradation pathways to protein turnover in various tissues (red and white muscle, liver, heart and gills). *K*_*S*_ was lower in the white muscle and in liver of the fasted fish compared to the fed fish. There were no observable effects of food deprivation on the protease activities in any of the tissues with the exception of liver, where the ubiquitin proteasome pathway seemed to be activated during fasting conditions. Lysosomal proteolysis appears to be the primary degradation pathway for muscle protein, while the ubiquitin proteasome pathway seems to predominate in the liver. We speculate that Arctic charr regulate protein metabolism during food deprivation to conserve proteins.

## Introduction

Animal growth depends on the opposing metabolic processes of protein synthesis (*K*_*S*_) and degradation [1, 2]. Soft tissue growth is produced through retention of a portion of synthesized proteins [2], a process well characterized due to the advances in practical methodologies [2–6]. In contrast, protein degradation, which allows individuals to recycle damaged or exogenous proteins, is poorly characterized [2].

Three of the most important protein degradation pathways are the ubiquitin proteasome pathway, lysosomal cathepsins and the calpain system [7–9]. The ubiquitin proteasome pathway predominates in mammals. Damaged proteins are targeted for degradation via covalent binding of ubiquitin to the protein substrate in an ATP-requiring reaction [7, 10]. The ubiquitin chains that form on protein substrates are recognized and degraded by a protease complex, the 26S proteasome [7]. Lysosomal vacuoles have a high amount of cathepsin proteinases with a wide range of specificities [11]. Calpains are a family of Ca^+^ dependent proteases found in mammals [8, 12] and fish [13–15] that cleave hundreds of known proteins, some of which are involved in signal transduction [8].

Dynamics of protein metabolism reflect changes in biotic and abiotic conditions (reviewed in [2, 3]). For example, food availability affects the ubiquitin proteasome pathway in fish [16–18] and *Sepia officinalis* [19]. Temperature influences the ubiquitin proteasome pathway in spotted wolfish (*Anarhichas minor*) [20, 21]. Lysosomal protein degradation responds to starvation in mammals and salmonids [9, 22], and calpains are activated by fasting or nutritional status in mammals [8, 12] and fish such as sea bream (*Sparus aurata*) [23]. The present study investigates the effects of a 36-day food deprivation period on these aspects of protein metabolism in tissues of Arctic charr (*Salvelinus alpinus*).

As the world’s northernmost freshwater fish species, Arctic charr experience large variations in environmental conditions, which contribute to seasonal variations in their growth rate [24]. They are exposed to long dark winters with little food availability, a time during which they experience negative growth, and short summers during which they replenish depleted energy reserves [24, 25]. Three distinct phases characterize the typical physiological responses to food deprivation in most vertebrates and invertebrates [26, 27]. The first few days of deprivation, phase I, is a transient phase during which carbohydrates, lipids and proteins are used to maintain basal metabolism. During phase II, the animals mobilize primarily lipid reserves with low rates of protein catabolism [26, 27]. Once lipid depletion reaches a critical threshold, animals enter phase III in which, proteins are oxidized as a fuel of last resort. Only then, are the animals considered to be in a true phase of starvation [28, 29]. While many animals experience these three phases, many studies have found variations in physiological responses to food deprivation in different species or even in populations within the same species [30–32].

This study presents, for the first time, the fractional rate of protein synthesis (*K*_*S*_) and the activity of several proteases in tissues of fed and food-deprived fish. By measuring both *K*_*S*_ and protease activities we are able to better understand the adjustments of protein metabolism during prolonged fasting periods. After 36 days of food deprivation, *K*_*S*_ is markedly decreased in the muscle and liver, while it is maintained in the heart and gills. Overall, there was no observable activation of protein degradation, indicating that the fish resisted entering in the third phase of starvation after 36 days of fasting.

## Materials and Methods

### Animals

Arctic charr (Fraser strain) were obtained from the Coastal Zones Research Institute Inc., (Shippagan, NB, Canada). Eighty fish were held at the Université de Moncton, NB, Canada from January to April 2014. Fish were held in a 1,200 L tank equipped with a freshwater recirculation system. The fish were fed a commercial salmon diet (Ewos Transfer, Ewos Canada, Surrey, BC) at 4% body weight per day, every other day. Water temperature was maintained at 12°C and fish were exposed to natural photoperiod. At the end of March, 20 fish were semi-randomly selected (avoiding the runts), weighed and transferred into two experimental tanks of 100 L (n = 10 per tank, density of approximately 25 kg/m^3^). One group of fish was deprived of food for a period of 36 days while the second group was continued on the commercial salmon diet at 4% body weight per day, every other day. After the starvation period, the rate of protein synthesis was measured in individual fish of both groups. Fish sampling was conducted approximately 28 hours after the fed group’s last meal to avoid the absorptive stage. The fish were then anesthetised in 50 mg/L of benzocaine and killed by a blow to the head. Samples of red and white muscle, liver, heart and gills were collected for each fish and placed immediately in liquid nitrogen. During sampling, the guts were inspected to ensure that the intestines were empty in order to confirm that the fish were in a post-absorptive stage. Samples were stored at -80°C until further analyses. Growth trajectories were estimated using the average mass of the ten fish in each group before and after the starvation period. The protocol was approved by the animal care committee of Université de Moncton.

### Protein Synthesis

We measured *K*_*S*_ using the flooding dose technique of Garlick *et al*. (1980), which was modified following Lamarre *et al*. (2015) for use with stable isotopes instead of radioactive tracers [4,33]. The modified technique has been validated on Arctic charr using the same group of fish as outlined in a previous publication [33]. Prior to sampling, on day 36, fish received an intraperitoneal injection of a solution of 150 mM phenylalanine (PHE) containing 50% ring [D_5_]-L-phenylalanine ([D_5_]-PHE, 98%, Cambridge Isotope Laboratories, Inc. Andover, MA, USA) at a dosage of 1 ml per 100 g of body mass. Following an incorporation period of four hours (as per ref. [33]), fish were sampled as described above. The abdominal cavity was immediately exposed and rinsed with distilled water to wash off any unabsorbed tracer. The fractional rate of protein synthesis using [D_5_]-PHE was measured as in Lamarre *et al*. (2015) [33]. Approximately 75 mg of tissue were homogenized in 1 ml of 0.2 M perchloric acid (PCA) with a sonicating homogenizer (Q55 Sonicator, Qsonica Inc.) and centrifuged at 15,000 *g* for 5 minutes at 4°C. The supernatant containing the free-pool of amino acids was saved (frozen until further analyses) and the remaining protein pellets were washed (in 1 ml of 0.2 M PCA) and then centrifuged (5 minutes at 15,000 *g*) three times. The pellets were further washed with acetone to remove lipids before being hydrolyzed in 6 M HCl at 110°C for 18 hours. PHE was extracted from the hydrolyzed protein-pool and the free-pool samples by solid phase extraction using C18 cartridges (Bond-Eut-C18, 100 mg, 1 ml, Varian Inc.). The extracted amino acids were then dried by heating the tubes at 110°C for an hour. PHE was derivatized via an alkylation procedure using pentafluorobenzyl bromide as follows. The dried samples were solubilized in 75 μl of distilled H_2_O and 50 μl of this sample solution was then transferred into GC-MS vials containing 20 μl of phosphate buffer (0.5 M, pH 8.0) and 130 μl of pentafluorobenzyl bromide (PFBBr) solution in acetone. [D_5_]-PHE specific enrichment of the free-pool and the protein-pool were determined by GC-MS analyses. The analyses were performed using an Agilent gas chromatograph (model 6890N) interfaced with a single quadrupole inert mass selective detector (MSD, model 5973). The chromatographic column was a Zebron ZB-5MS Capillary GC Column 30 m x 0.25 mm x 0.30 μm (Phenomenex inc.). The injector was operated in pulsed splitless mode with a valve off-time of 1.5 min. The injector, transfer line and ion source were kept at 250°C. Helium was used as the carrier gas at a constant flow rate of 0.9 ml·min^-1^. The GC conditions were as follows: initial oven temperature was 70°C and the temperature was increased to 300°C at a rate of 25°C·min^-1^. The temperature was then held at 325°C for 10 min (program run time was 20 min). The mass spectrometer was operated in selected ion monitoring (SIM) with mass-to-charge ratio (m/z) 300 and m/z 305 for PHE and [D_5_]-PHE, respectively. The peak detection and integration were performed using MSD Chemstation (D.01.00 Build 75, Agilent). The fractional rate of protein synthesis (*K*_*s*_%·day^-1^) was calculated using the following Eq 1:

$$K_{S}\;=\frac{S_{b}}{S_{a}}\times \frac{1440}{t2-t1}\times 100,$$ (1)

where *S*_*b*_ is the enrichment of the protein-pool (*S*_*b*_ = [D_5_]-PHE /(PHE + [D_5_]-PHE)) and *S*_*a*_ is the enrichment of the free amino acid pool (*S*_*a*_ = [D_5_]-PHE /(PHE + [D_5_]-PHE)), *t* is the incorporation time and 1440 is the conversion from minute to day (modified from [4, 33]). In cases where the PHE free-pool enrichment decreased over time, we used an alternate model to calculate *K*_*s*_ (Eq 2);

$$K_{S}\;=\frac{S_{b(t2)}-S_{b(t1)}}{S_{a(t2-t1)}}\times \frac{1440}{t2-t1}\times 100,$$ (2)

where *S*_*b*_(t2) is the final protein-bound [D_5_]-PHE and *S*_*b*_(*t*1) is the average incorporation at an earlier time (in this case; 60 min). *S*_*a*_(*t*2-*t*1) is the average enrichment in the free-pool between time 2 (*t*2) and time 2 (*t*1) [4,33,34].

### 20S proteasome activity

In order to measure the maximal chymotrypsin-like activity of the 20S proteasome, tissues were homogenized in nine volumes of lysis buffer (50 mM Tris, 0.1 mM EDTA, pH = 8) using a sonicating homogenizer (Q55 Sonicator, Qsonica Inc.), and centrifuged at 13,000 *g* for 60 minutes at 4°C. Protein concentration of the supernatant was determined with a Bradford protein assay kit (Bio-rad, Hercules, CA) [35]. The 20S proteasome activity was measured using a modified version of the methods used by Shibatani and Ward (1995) [36]. Fifty μg of sample protein was placed in wells of a black-bottomed 96-well plate in quadruplicates along with 100 μl of assay buffer (100 mM Tris, 0.0475% SDS, pH = 8) and 10 μl of fluorogenic substrate LLVY-AMC (Enzo Life Sciences, Inc., Burlington, ONT, p802-0005; 400 μM in Tris buffer). Finally, an inhibitor solution of the 20S proteasome (ZLLL-CHO, Enzo Life Sciences, Inc., Burlington, ONT, PI102-0005) was added to the fourth replicate of each sample. Fluorescence was read continually at excitation/emission wavelengths of 370/430 nm with a multimode plate reader (Varioskan Flash, Thermo Fisher Scientific, Inc., Waltham, MA USA). The inhibitor-sensitive activity of the 20S proteasome was calculated and expressed using arbitrary fluorescence units per minute, per 50 μg of protein.

### Levels of polyubiquitinated proteins

Levels of polyubiquitinated proteins were assessed using dot blot analyses [19]. Aliquots from the tissue homogenates used for 20S proteasome activity were also used for dot blot analyses. For each sample, 25 μg of protein was spotted on a nitrocellulose membrane. The membrane was first blocked with 5% BSA and levels of polyubiquitinated proteins were detected using a specific antibody (Polyubiquitinated conjugates, mAB, FK1, Enzo, BML-PW8805). Dots were made visible by enhanced chemiluminescence (ECL), using a horseradish peroxidase (HPR) conjugated antibody (ab97230, Abcam, Cambridge, MA). The membranes were imaged using a Chemidoc MP (Bio-Rad, Hercules, CA) and the dots’ intensity was evaluated using Image Lab 5.1 (Bio-rad, Hercules, CA).

### Protease activity

Calpain and cathepsin maximal activity was measured using a fluorescent protein substrate. BODIPY-FL-conjugated casein was prepared and used as a substrate for calpain and cathepsins [37]. We homogenized the tissues as described above; however, the homogenate was centrifuged at 13,000 *g* for 10 minutes instead of 60 minutes. Protein concentration of the supernatant was again determined with a Bradford protein assay kit (Bio-rad, Hercules, CA) [35]. Cathepsin activity was determined at pH 2.5 (cathepsin D- and E-like; 43.5 mM citric acid, 112 mM Na_2_HPO_4_, 10 mM DTT), and pH 5.5 (cathepsin A- B- H- and L-like; 97 mM citric acid, 5.8 mM Na_2_HPO_4_, 10 mM DTT). Muscle calpain activity was measured in an assay buffer containing 20 mM Tris, 1 mM EDTA, 10 mM CaCl_2_, 100 mM KCl and 0.1% beta-mercaptoethanol, pH 7.5. To conduct the assays, 100 μl of sample homogenate and 100 µl of enzyme assay buffer containing 10 μg BODIPY-FL-casein were added to each well of a black-bottom 96 well plate. The fluorescence was then read for 15 minutes at excitation/emission wavelengths of 485/535 nm using a multimode plate reader (Varioskan Flash, Thermo Fisher Scientific, Inc., Waltham, MA USA). The enzyme activities were expressed in arbitrary fluorescence units·min^-1^·mg protein^-1^.

### Statistical analyses

A student’s t-test was used in order to compare mean body mass before and after the experiment in both fed and fasted groups. We compared fed and fasted fish within and between each tissue using a two-way ANOVA followed by a Bonferonni post-hoc analysis. A Box-Cox transformation was applied to the data whenever the heteroscedasticity and normality of the residuals criteria were not met. In order to compare capacity for protein turnover between tissues, we calculated ratios of the enzymatic activity for the different proteases relative to protein synthesis in each tissue, for each fish. These ratios were calculated by dividing the measured protease activity by *K*_*S*_ in the same tissue. We used a one-way ANOVA to compare the ratios among tissues, and Box-Cox data transformations was applied on the ratios when needed. Data Desk 6.3 (Data Description, Inc.) was used for the statistical analyses and Prism 5 (GraphPad Software Inc.) was used for the figures.

## Results

No mortalities were recorded during the experiment. Prior to the fasting period, both fed and unfed groups had similar average body masses. The average growth trajectory of fed Arctic charr was 0.74% · day^-1^ during the experimental period (t-test, p = 0.001). After 36 days of food deprivation, the mass of food deprived fish did not change (Table 1; t-test, p = 0.67). The average protein concentration in the various tissues did not differ between the fed and fasted group (Table 2).

**Table 1:** Initial mass (g), final mass (g) and specific growth rate (%·day^-1^) of Arctic charr following a 36-day fasting period. Values are expressed as means ± s.e.m. (n = 10).

	Fed	Fasted
**Mean initial mass (g)**	242.16 ± 47.26	272.54 ± 60.94
**Mean final mass (g)**	316.42 ± 86.36*	283.219 ± 50.4
**Growth trajectory (%·day**^**-1**^**)**	0.74	0.11

*Indicates significant difference between final and initial mass in either group

**Table 2:** Average protein concentration (mg · g of tissue^-1^) in various tissues of fed and fasted Arctic charr. Values are expressed as means ± s.e.m. (n = 7).

	Protein concentration (mg · g of tissue^-1^)	
Tissue	Fed	Fasted
White muscle	124.4 ± 5.9	119.4 ± 13.0
Red muscle	70.6 ± 6.5	64.7 ± 0.72
Liver	168.6 ± 9.2	184.4 ± 8.9
Heart	91.2 ± 5.5	88.3 ± 6.9
Gill	92.9 ± 6.8	96.5 ± 5.0

### Protein synthesis

**Fig 1:**
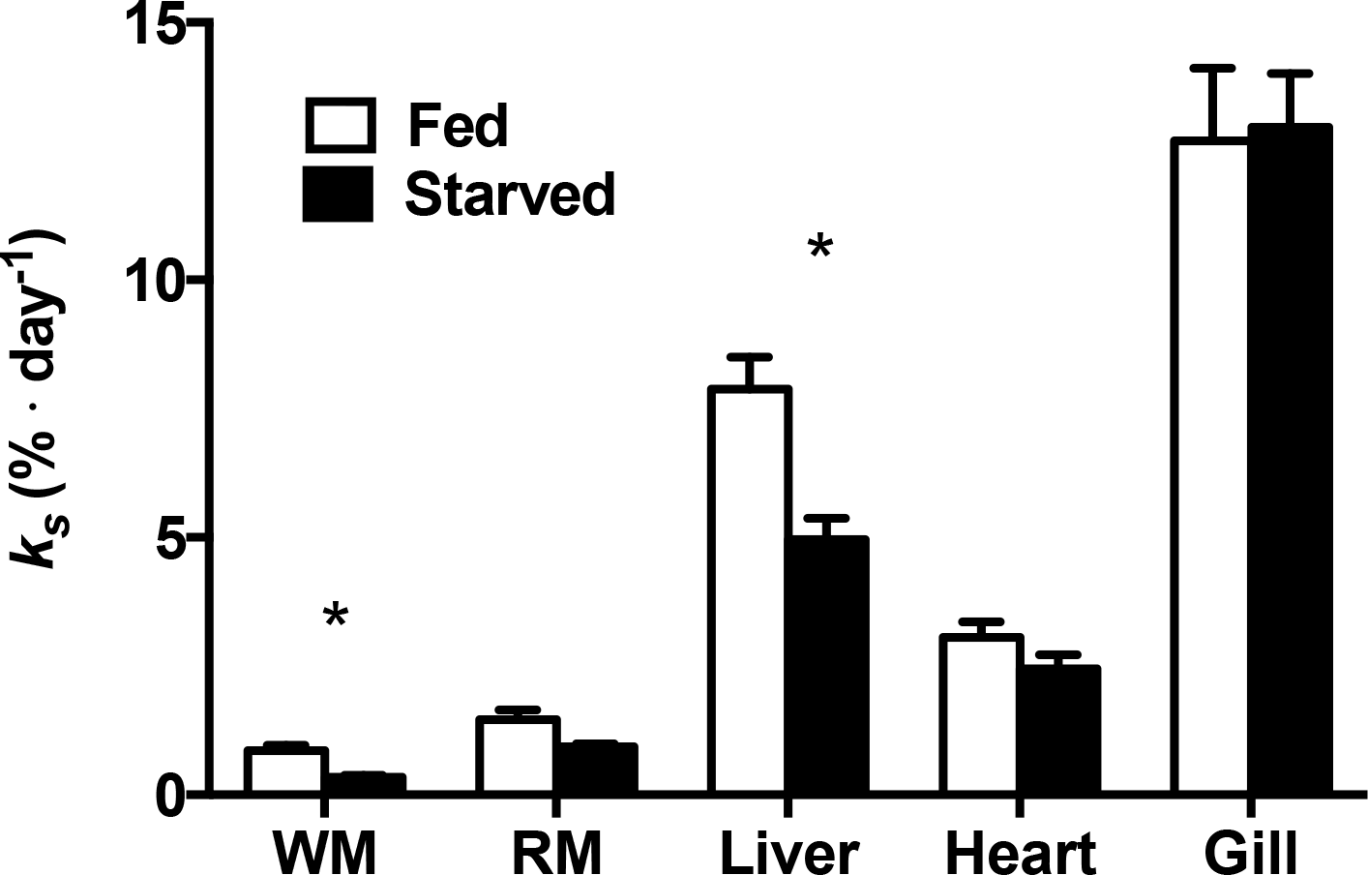
Fractional rate of protein synthesis (*K*_*S*_;%·day^-1^) of white muscle, red muscle, liver, heart and gill of fed (white) and 36 days fasted (black) Arctic charr. * Indicates a significant difference between treatments (p < 0.05). Values are expressed as means ± s.e.m. (n = 10).

The response of *K*_*S*_ to fasting varied among tissues (ANOVA, p = 0.031). *K*_*S*_ decreased significantly in the white muscle and liver of fasted fish compared to the fed group but not in the red muscle, heart or gills (Fig 1). *K*_*S*_ decreased by more than half in the white muscle (ANOVA, p = 0.003) and approximately one third (ANOVA, p ≤ 0.001) in the liver when compared to fed fish. Tissues varied in the levels of *K*_*S*._ The relative tissue levels were highest in the gills in both the fed and fasted groups, liver showed the second highest, while muscles had the lowest *K*_*S*_.

### Protein degradation

**Fig 2:**
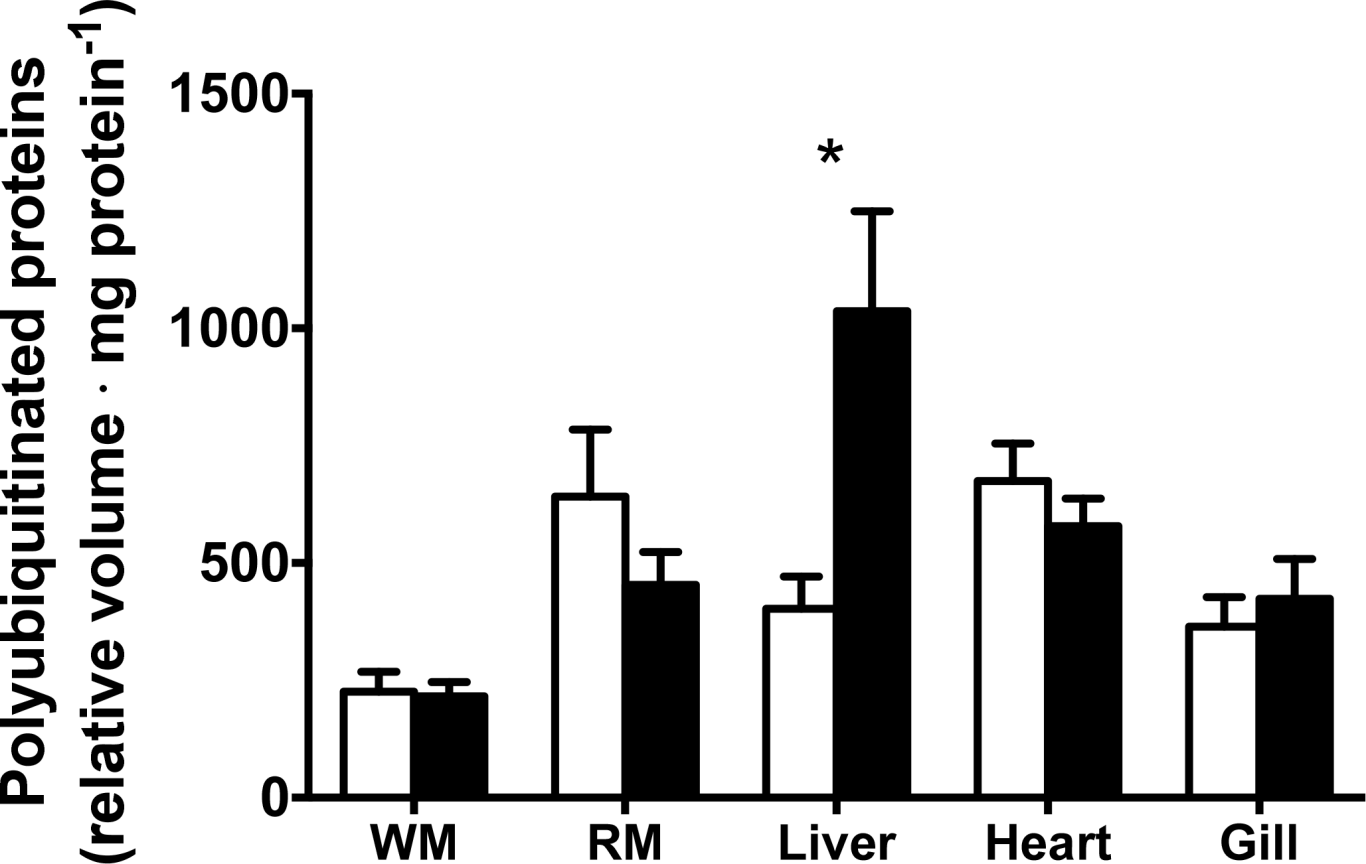
Levels of polyubiquitinated proteins in tissues of fed and fasted Arctic charr (white and black, respectively). * Indicates a significant difference between the fed and fasted groups. Values are expressed as means ± s.e.m. (n = 7).

Food deprivation led to increased activity of some components of the ubiquitin proteasome pathway in the liver only (Table 3). More specifically, there was significantly higher 20S proteasome activity (p = 0.042) and almost twice as many polyubiquitinated proteins (p = 0.002) in the liver of fasted fish compared to the fed group (Fig 2). The concentration of polyubiquitinated proteins was greatest in the liver, and 20S proteasome activity was greatest in liver and gills. After 36 days of fasting, there was no difference in activity of the other proteases measured when compared to the fed group (Table 3).

**Table 3:** 20S proteasome, cathepsin pH 2.5, pH 5.5 and calpain activity in white muscle (WM), red muscle (RM), liver, heart and gill of fed and fasted Arctic charr. Activities are expressed in fluorescent units per mg protein per minute (means ± s.e.m., n = 7). *Indicates a significant difference between fed and fasted groups.

	20S proteasome		Cathepsin pH 2.5		Cathepsin pH 5.5		Calpain	
Tissue	Fed	Fasted	Fed	Fasted	Fed	Fasted	Fed	Fasted
**WM**	0.46 ± 0.02	0.46 ± 0.03	1.63 ± 0.11	2.20 ± 0.45	6.24 ± 0.37	7.88 ± 1.57	2.64 ± 0.25	3.11 ± 0.56
**RM**	3.62 ± 0.23	2.93 ± 0.47	3.87 ± 0.60	4.99 ± 1.15	13.04 ± 1.38	17.98 ± 4.18	8.76 ± 2.16	10.40 ± 3.25
**Liver**	10.61 ± 0.94 **	14.03 ± 1.10 **	2.33 ± 0.20	2.98 ± 0.19	5.31 ± 0.40	4.14 ± 0.28	n.d.	n.d.
**Heart**	5.98 ± 0.33	3.53 ± 0.45	6.45 ± 0.15	6.79 ± 0.20	8.36 ± 1.22	8.47 ± 0.65	n.d.	n.d.
**Gill**	12.43 ± 0.79	11.71 ± 1.00	5.01 ± 0.47	5.45 ± 0.46	9.82 ± 1.77	9.31 ± 0.99	n.d.	n.d.

n.d. not determined

** Indicates a significant difference betweem fed and fasted fish;

### Preferred pathways of protein degradation

**Fig 3:**
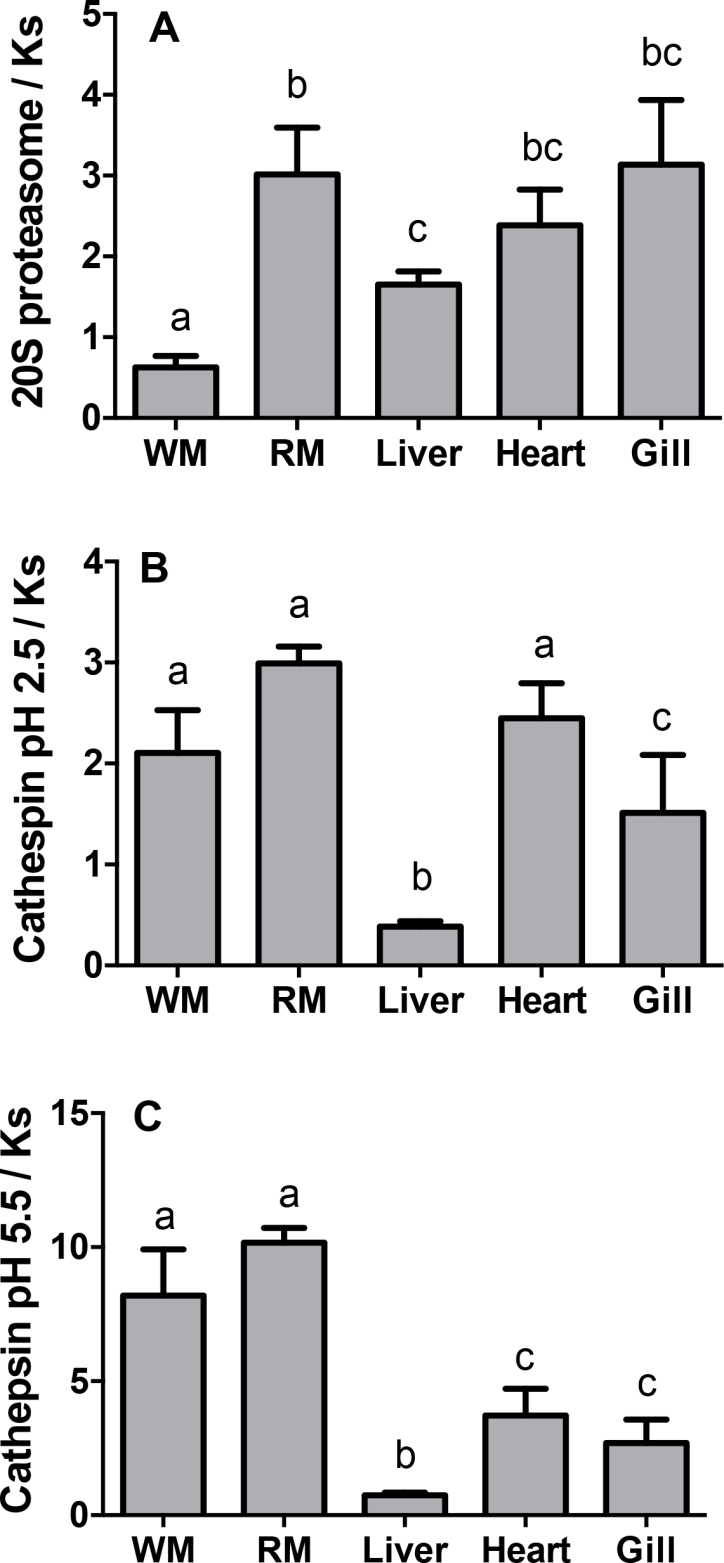
Ratios of protease activity relative to fractional rates of protein synthesis (*K*_*s*_) in tissues of fed Arctic charr. (A) maximal 20S proteasome activity, (B) cathepsin pH 2.5 activity and (C) cathepsin pH 5.5 activity. Note that these ratios do not have units and are for comparison purposes only. Different letters indicate significant difference (p < 0.05). Values are expressed as means ± s.e.m. (n = 7).

In white muscle, the ratio of 20S proteasome activity to *K*_*S*_ was significantly lower than in the other tissues (Fig 3A, p ≤ 0.001). This ratio was elevated in the red muscle and intermediate in the liver and other tissues. The ratio of cathepsin activity to *K*_*S*_ was generally higher in both red and white muscle, intermediate in heart and gills and lowest in liver (Fig 3B and 3C, p ≤ 0.001).

## Discussion

A 36-day fasting period led to decreased growth rates, but not weight loss, in our fasted fish compared to the fed fish. Given Arctic charr’s ability to tolerate starvation during several months, one month of food deprivation was likely insufficient to stimulate weight loss. In general, fish are able to tolerate long periods without food, and some do not lose weight even during periods of prolonged starvation [28, 38]. In addition, water accumulation in the tissues and organs can mask the loss of lipids and proteins during food deprivation when measuring the mass of live animals [28, 39, 40]. For example, after eight and twelve weeks of food deprivation, there is a proportional increase in organ water content as lipid content decreases in Arctic charr [41]. It is possible that water content increased in tissues following the fasting period, thus contributing to the apparent, albeit marginal gain in mass of the fasted fish. This increase in water content likely does not result from a displacement of proteins but from a displacement of lipids, because protein concentration in the tissues did not differ between the fed and fasted fish.

### Protein synthesis

Metabolically active tissues typically had the highest *K*_*S*_, which ranked from highest to lowest in the following order: gill, liver, heart, red muscle and white muscle. These rates and ranks generally correspond with information in the literature (reviewed in [2]). Typically, during periods of food deprivation, rates of protein synthesis slows in fish [42, 43], which was observed in the liver and muscle of fasted fish in this study. White muscle protein synthesis is commonly used as an indicator of whole-animal growth since it makes up more than half of the fish’s body mass [44]. Indeed, the decrease of white muscle *K*_*S*_ in response to food deprivation in this study is consistent with the view that this tissue is very sensitive to changes in diet [18, 20, 45]. *K*_*S*_ also decreased in the liver but not in the heart or gills, suggesting that the latter tissues are protected during fasting [46].

### Protein degradation

To our knowledge, we are the first to measure the activity of various proteases in fasting Arctic charr. We chose to express protease activity relative to protein concentration, as the use of protein concentration as a denominator could skew the results if the tissue protein concentration changed during the experiment. As shown in Table 2, the protein concentration of the various tissues did not differ between fed and fasted fish.

With the exception of an increase in the ubiquitin proteasome pathway in the liver of fasted fish, 36 days of food deprivation had no effect on the activity of the proteases measured. This is in contrast to the response of rainbow trout (average body mass; 36.0 g ± 1.9 SEM) which downregulated the ubiquitin proteasome pathway in the liver and white muscle by decreasing 20S proteasome activity and the concentration of polyubiquitinated proteins in response to two weeks of fasting [17]. However, the Arctic charr in the present study were similar to rainbow trout (average body mass;185.5 g ±10.9 SEM after two weeks of food deprivation), in terms of the lack of response of cathepsin activity in the liver to feed deprivation [47]. Our findings are also consistent with those of Salem *et al*. (2007) which found a significant effect of food deprivation on activity of the 20S proteasome pathway but not of cathepsins in the liver of rainbow trout (average body mass; 193 g ± 15.7 SEM) after three weeks of food deprivation [48].

Muscle proteins are typically regarded as the primary source of amino acids for the metabolically active tissues during periods of food deprivation [45, 49]. Accordingly, we expected to see an increase in indicators of protein degradation in the muscle of fasting fish. However, we detected no changes in the 20S proteasome, cathepsin and calpain activities in the muscle of Arctic charr after 36 days of fasting. This is in contrast to the response of expression of the ubiquitin proteasome pathway, as well as lysosomal cathepsins and calpains in the muscle after two weeks of starvation in rainbow trout [50], and increase in cathepsin activity in muscle of sockeye salmon (*Oncorynchus nerka*) and Atlantic cod (*Gadus morhua*) starved for 12 weeks [49, 51]. There are contradictory studies in the literature where calpain mRNA expression decreased in muscle during starvation in one study [50] whereas their activity and expression increased in another [52].

The lack of alterations of protease activities in the muscle of fasted fish in our experiment may indicate that the fasting period was not long enough for the fish to deplete their lipid stores and switch to protein-dominated catabolism for energy production. The discrepancies among previous studies and this experiment may also be due to major differences in the experimental conditions such as length of food deprivation period, size and age of fish, species, temperature, and other environmental conditions, which influence the fish’s response to starvation [53].

### Preferred pathways of protein degradation

While there are no techniques available for measuring the rate of protein turnover in tissues of living fish, it is useful to compare the maximal activities of enzymes involved in protein degradation among tissues. For instance, the chymotrypsin-like activity of the 20S proteasome is much lower in white muscle than it is in the liver, while the cathepsin activity at pH 5.5 is slightly higher in muscle. However, these direct comparisons would only be relevant in terms of protein metabolism if *K*_*S*_ were the same in the tissues we wish to compare. Thus we normalized the protease activities by examining the ratios of the protease activities relative to *K*_*S*_ for every tissue. The ratio units are arbitrary as *K*_*S*_ is measured *in vivo*, while the protease activities are measured *in vitro* as maximal activity, at room temperature and over a different time scale. Nevertheless, since the enzyme assays and *K*_*S*_ measurements were conducted in identical conditions among tissues (dilutions, time and temperature), we can compare the capacity for protein turnover via the different protein degradation pathways relative to *K*_*S*_ between tissues. A greater ratio indicates a greater capacity for protein degradation per unit of protein synthesis. Since the fasting period predominantly affected *K*_*S*_, we did not compare ratios between the fed and fasted fish because the different *K*_*S*_ would drive the observed differences between the ratios. Protein degradation via lysosomal cathepsins, especially those active at pH 5.5, are predominant in red and white muscles compared to other tissues and the ubiquitin proteasome pathway seems less important in the white muscle. This observation mirrors the results of Seiliez *et al*. (2014) working on isolated rainbow trout myotubes [54]. It is remarkable that the liver appears to have little capacity for protein degradation relative to its rate of protein synthesis when compared to the other tissues measured. This may be related to the major role of liver as a protein-secreting organ. Proteins such as albumin are synthesized in the liver then excreted in the plasma and later mostly degraded in other tissues [55].

These protease activities relative to *K*_*S*_ are indicative of the maximum capacity for protein turnover via these pathways rather than actual turnover. Still, our results suggest that there are large variations in the relative importance of each protein degradation pathway among different tissues. This information further supports the recent view that lysosomal protein degradation plays a major role in protein turnover in the fish muscle [54]. The modulation of this pathway via manipulation of the dietary amino acid profile may help to improve muscle protein retention in fish [54].

### Conclusion

To our knowledge, this is the first study to measure *K*_*S*_ and the activity of proteases simultaneously in tissues of the same fish during prolonged fasting. We were also able to evaluate the capacity for protein turnover via different degradation pathways in fed fish. This information is important for understanding the dynamics and control of protein metabolism on fish growth. We demonstrate that after 36 days of food deprivation, there is no detectable loss of total body mass and muscle protein in Arctic charr, an atypical response for fish after more than one month of fasting. Arctic charr did not catabolise their muscle proteins for metabolic energy during this period of fasting. A finding in contrast with the closely related species rainbow trout. The degradation pathways of the latter species have been altered in response to two and four weeks of food deprivation [17, 50]. Arctic charr likely evolved numerous adaptive strategies to cope with cold temperatures, reduced day length and long periods of limited food availability without using their muscle proteins for fuel [56].

It is important to characterize the adjustments in protein metabolism to understand the regulation of growth, and therefore to assess the nutritional status of captive or wild fish. This research provides some insight into the strategies used to tolerate natural periods of food deprivation in anadromous coldwater fish species. Further investigations are warranted to elucidate the sequence of molecular events that are involved in the onset of protein degradation during food restriction. Given their generally high tolerance to food deprivation, fish provide us with a valuable model to study the adjustments of protein metabolism during catabolic conditions.
